# Genetic data from algae sedimentary DNA reflect the influence of environment over geography

**DOI:** 10.1038/srep12924

**Published:** 2015-08-11

**Authors:** Kathleen R. Stoof-Leichsenring, Ulrike Herzschuh, Luidmila A. Pestryakova, Juliane Klemm, Laura S. Epp, Ralph Tiedemann

**Affiliations:** 1 Alfred Wegener Institute Helmholtz Centre for Polar and Marine Research, Periglacial Research, Telegrafenberg A43, 14473 Potsdam, Germany; 2 University of Potsdam, Institute of Biochemistry and Biology, Unit of Evolutionary Biology/Systematic Zoology, Karl-Liebknecht-Strasse 24-25, 14476 Potsdam, Germany; 3 North-Eastern Federal University of Yakutsk, Department for Geography and Biology, ul. Belinskogo 58, 677000 Yakutsk, Russia; 4 University of Potsdam, Institute of Earth and Environmental Science, Karl-Liebknecht-Strasse 24-25, 14476 Potsdam, Germany

## Abstract

Genetic investigations on eukaryotic plankton confirmed the existence of modern biogeographic patterns, but analyses of palaeoecological data exploring the temporal variability of these patterns have rarely been presented. Ancient sedimentary DNA proved suitable for investigations of past assemblage turnover in the course of environmental change, but genetic relatedness of the identified lineages has not yet been undertaken. Here, we investigate the relatedness of diatom lineages in Siberian lakes along environmental gradients (i.e. across treeline transects), over geographic distance and through time (i.e. the last 7000 years) using modern and ancient sedimentary DNA. Our results indicate that closely-related *Staurosira* lineages occur in similar environments and less-related lineages in dissimilar environments, in our case different vegetation and co-varying climatic and limnic variables across treeline transects. Thus our study reveals that environmental conditions rather than geographic distance is reflected by diatom-relatedness patterns in space and time. We tentatively speculate that the detected relatedness pattern in *Staurosira* across the treeline could be a result of adaptation to diverse environmental conditions across the arctic boreal treeline, however, a geographically-driven divergence and subsequent repopulation of ecologically different habitats might also be a potential explanation for the observed pattern.

DNA-based investigations have confirmed the existence of biogeographic patterns in eukaryotic microbes with high dispersal potential and putatively cosmopolitan distribution^1^,^2^,^3^. The patterns detected are presumed to be established by geographic constraints, environmental conditions^3^,^4^,^5^, or a coupling of variables. The underlying processes that initiate, distribute and maintain diversity are usually inferred based on the analyses of recent spatial data^6^. Information from the past is rarely available, but its inclusion can help to understand the established local to global biogeographic patterns^7^,^8^,^9^,^10^,^11^. Indeed, the analyses of taxonomic turnover through historical environmental changes at a single location would be appropriate to test the influence of ecological factors regardless of geographic constraints. Ancient DNA studies on environmental samples can directly address past genetic diversity, and investigate taxonomic turnover in a single locality in relation to environmental change^12^,^13^,^14^,^15^. Beyond unravelling past genetic diversity, the use of phylogenetic informative markers can facilitate the analysis of evolutionary relatedness between lineages extracted from (old) environmental DNA^15^,^16^. Until now, the combination of ancient environmental DNA and the analysis of evolutionary relationships between closely-related lineages has not been considered in the context of biogeographic patterns in eukaryotic microbes.

Ecotones—ecological transitional zones along a steep environmental gradient—are predestined to disentangle geographically- and/or ecologically-driven processes^17^. The boreal treeline is one of the most extensively investigated ecotones characterized by a gradual change of vegetation from treeless tundra to forests, which are, in Siberia, solely formed of larch trees (*Larix* sp.). Temporal changes of the treeline on millennial timescales are well documented and reflected in pollen analyses^18^,^19^. Generally, documented changes in vegetation across the Siberian treeline can be considered as a proxy for related changing variables, such as temperature and lake-water chemistry^20^, which in turn can affect aquatic communities in the embedded waters, for example diatom species composition, such as has been observed in arctic Lapland lakes^21^.

A prevalent and diverse group of diatoms in the abundant thermokarst lakes of the Siberian treeline ecotone is *Staurosira* and related taxa^22^,^23^, and their spatial turnover is known to be correlated with changing environments across this ecotone^16^,^20^,^24^. Phylogenetic relationships within this group have recently been reassessed using molecular genetic data, and due to a strong evolutionary relationship between the morphologically similar genera *Staurosira*, *Staurosirella* and *Pseudostaurosira*, a taxonomic integration into a single genus *Staurosira* has been suggested^25^. Taxonomic assignment using DNA sequences is currently becoming more widespread, and the plastid gene of the large subunit of the 1,5 bisphosphate carboxylase/oxygenase (*rbcL*) has proven to be particularly suitable for detecting both intra- and inter-specific variants and cryptic speciation in diverse diatom genera^17^,^26^. Environmental DNA analysis^27^ offers the possibility of retrieving genetic data directly from environmental samples^15^,^16^,^28^,^29^, but the degraded nature of DNA restricts the analysis to fragments of short length and markers present in multiple copies, e.g., plastid genes^30^,^31^. A short fragment of the *rbcL* marker has proven useful for analyses of environmental samples, such as sedimentary DNA (*sed*DNA)^15^,^16^. In a previous study, this marker deciphered intra/interspecific diversity and the evolutionary relationships of *Staurosira* lineages across the treeline ecotone^16^.

Here, we investigate the spatial distribution and the evolutionary relationships of modern *Staurosira* lineages from lakes along three transects across the vegetation gradient of the Siberian treeline. Furthermore, we assess the temporal changes of *Staurosira* lineages and their evolutionary relationships in a sediment core through periods of historical vegetation change at the treeline. The temporal approach offers a way to investigate lineages’ turnover (in terms of assemblage and relatedness of lineages) through environmental changes regardless of the influence of geographic distance. Our survey uses a molecular group-specific genetic approach on modern and ancient *sed*DNA, further supported by microscopic inspection to investigate the mechanisms (environment or geography) responsible for local biogeographic and temporal patterns in diatoms across the treeline. Similar to phylogenetic community analyses in plant species^32^,^33^, our approach tests for the evolutionary relatedness of diatom lineages detected across the treeline and through time in order to distinguish between environmentally- and geographically-driven distribution patterns.

We hypothesize that if the biogeographic pattern in closely-related lineages of *Staurosira* is driven by geographic distance, we would expect to see a higher degree of relatedness among *Staurosira* lineages within geographic areas, and would not expect changes within a single sediment core through times of environmental change. If the establishment is linked to environmental change, rather than to geographic constraints, we would expect to see a high degree of relatedness among *Staurosira* lineages within similar vegetation types, and this association should be stable through time. We would therefore expect to see a turnover in *Staurosira* lineages contemporaneous with environmental change in the dynamic treeline area.

## Results

### Localities

**Figure 1:**
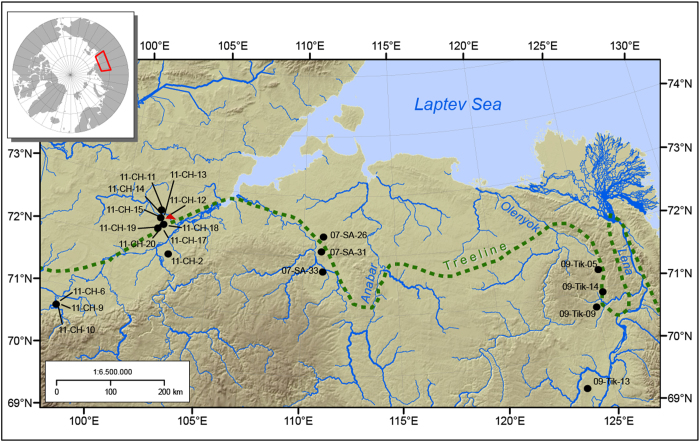
Study area in Siberia, Northern Russia. Black dots indicate the sampling sites of surface sediments and the red triangle indicates the position of the sediment core 11-CH-12A. This map was created with Esri Arc GIS Version 10.2.

The geographic positions of the surveyed lakes across the Siberian treeline and the coring locality are mapped in Fig. 1. Their physical and hydrochemical characteristics and vegetation-based parameters are given in Table 1.

### Primer specificity and genetic diversity

The *in silico* PCR approach revealed a high specificity to diatoms for *rbcL_194* and *rbcL_76* primers, as about 90% of the *in silico* amplificates were diatoms. The *in silico* PCR for the nested primer pairs (*rbcL_191* and *rbcL_67)* indicated less specificity to diatoms (50 to 70% of *in silico* amplified sequences were diatoms) (Supplementary Table S2). Primers amplifying the *rbcL_194* and *rbcL_191* fragments were specifically designed for *Staurosira* species and all known sequences of these taxa were among the *in silico* products. *In vitro* amplifications revealed that 74% of all obtained sequences types (=83% of all clones) were assigned to the targeted taxa. According to the guidelines of our verification protocol, we considered 23 sequence types belonging to *Staurosira* lineages as being authentic (from a total of 333 clones). These lineages were numbered from one to twenty-three (*191_01* to *191_23*). Primer combinations for the shorter *rbcL* fragments, *rbcL_76* and *rbcL_67*, were designed for the amplification of various diatom species and proved to be successful markers for core sediment applications^15^,^16^. To compare *Staurosir*a lineages resulting from surface and core samples, we shortened the sequences of the *191_01–23* lineages to the length of *rbcL_67* amplicon and thus created a *rbcL_a67* dataset consisting of 14 lineages (a*67_01–14*) (Supplementary Table S3). From twenty cored sediments samples, we sequenced 939 clones and identified 39 different diatom-specific *rbcL_67* sequences, whereof 13 (583 clones) were assigned to *Staurosira* lineages and numbered from *c67_01* to *c67_07* and *c67_15* to *c67_20*. The first seven *rbcL_a67* and *rbcL_c67* lineages are identical. All subsequent analyses were performed with three defined *Staurosira* datasets (*191_01–23*; a*67_01–14; c67_01–07* and *c67_15–20*). All taxonomic identifications are based on nucleotide BLAST search. BLAST results for the defined datasets containing accession number, taxa name and sequence identities are given in Supplementary Table S4.

### Phylogenetic inferences

**Figure 2:**
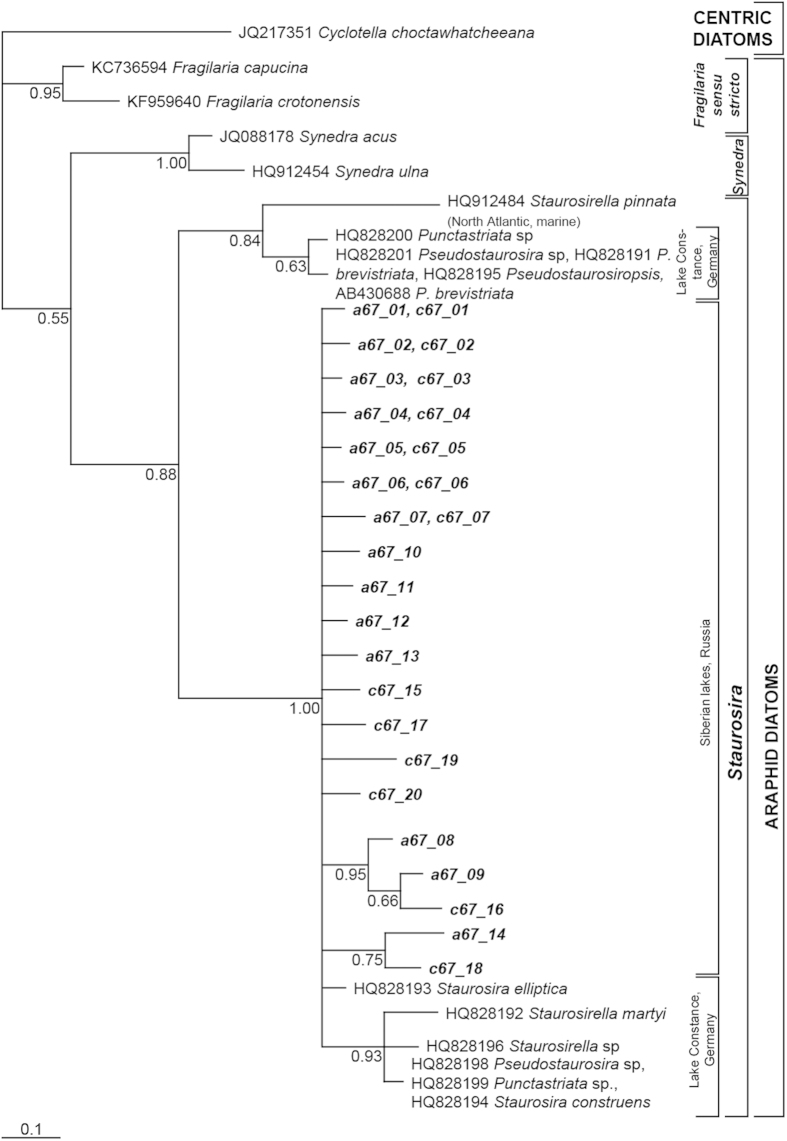


**Figure 3:**
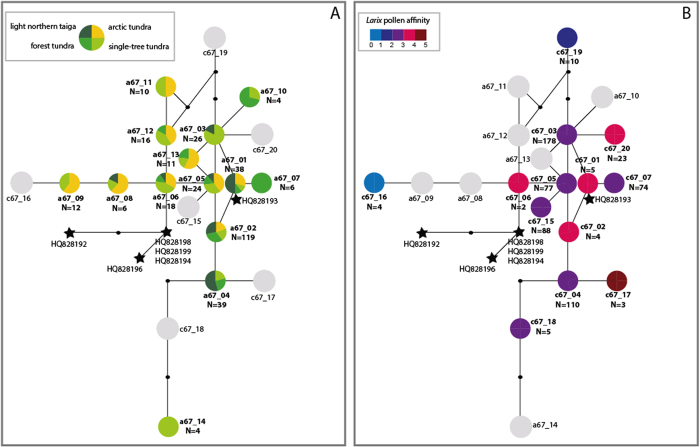
Haplotye network based on (**A**) *rbcL_a67* lineages (surface sediment) and *rbcL_c67* lineages (core sediment; coloured grey if they were not present in the surface sediment dataset) and (**B**) *rbcL_c67* lineages (core sediment) and *rbcL_a67* (surface sediment; coloured grey if they were not present in the core sediment data set) and six GenBank entries (indicated by a star: HQ828192 *Staurosirella martyi*, HQ828193 *Staurosira elliptica*, HQ828196 *Staurosirella* sp., HQ828198 *Pseudostaurosira* sp., HQ828199 *Punctastriata* sp., HQ828194 *Staurosira construens*) that clustered within the group of Siberian lineages (see Fig. 2). Coloured symbols show the proportion of each haplotype in the four vegetation types (**A**) or the *Larix* pollen affinity, which is defined by the mean *Larix* pollen percentage at which each lineage occurs (**B**) (see legend). Dots indicate missing haplotypes.

Phylogenetic trees for both datasets (*rbcL_a67/c67* (Fig. 2) and *rbcL_191* (Supplementary Fig. S2a)) indicate a clear separation of fragilarioid lineages, splitting monophyletic clades of *Fragilaria* sensu stricto (*Fragilaria* sp.) and *Synedra* from the *Staurosira/Staurosirella/Pseudostaurosira* cluster. This cluster is called “*Staurosira*” and contains two major clades, one clade that includes our *Staurosira* lineages obtained from Siberian lakes and lineages of *Staurosira, Staurosirella, Pseudostaurosira* and *Punctastriata* obtained from Lake Constance, Germany. The second clade consists of *Pseudostaurosira* isolates from Lake Constance, and a *Staurosirella pinnata* strain (HQ912484) of marine origin (North Atlantic). Additionally, we used haplotype networks of *rbcL_191* and *rbcL_67* lineages to gain a higher resolution of relationships within the *Staurosira* group. These networks provide information about the occurrence of Siberian *Staurosira* lineages in the four vegetation types (Fig. 3 and Supplementary Fig. S2b). The mean nucleotide distance between the *rbcL_67* lineages is 3.4 substitutions out of 67 nucleotides (5%) and the maximum nucleotide distance among lineages is six polymorphisms (9.8%). *rbcL_191* lineages have on average 7.4 (3.9%) and at maximum 15 (7.8%) nucleotide differences.

### Genetic data vs. environmental variables

NMDS analyses indicate a stronger correlation between modern *rbcL_a67* and *rbcL_191* lineages and vegetation than geographic distance (transects) (Supplementary Fig. S3). Stress values are relatively low in both datasets indicating that multidimensional data can be plotted in a two-dimensional graph, because only a few environmental variables explain the variation in the datasets. RDA results show a significant correlation between lineages’ occurrence and vegetation whereas geographic distance (transects) shows no significant correlation with the data (Supplementary Table S5).

**Figure 4:**
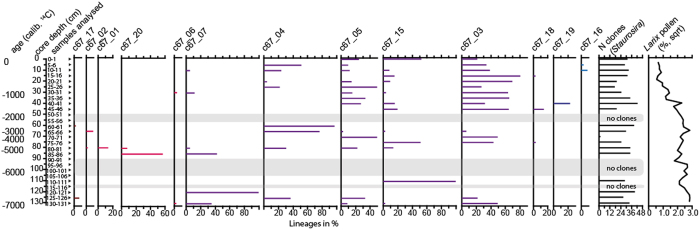
Down-core variations (11-CH-12A): Calibrated ^14^C ages, core depth in cm, relative frequencies of the *rbcl_c67* lineages (black arrows indicate the genetically analysed core samples) and *Larix* pollen percentage (data square-root transformed) of all terrestrial pollen grains. Lineages are grouped according *Larix* pollen affinity.

We defined the evolutionary relatedness between the lineages using patristic distances and used dbRDA to test for a correlation between lineages’ evolutionary relatedness and vegetation, and similarly with geography. Both datasets reveal a significant correlation with vegetation, but no (or only very weak) correlation with geography (Table 2). Accordingly, our results suggest that environmental variables, such as vegetation, rather than geographic distance, mainly impact the spatial genetic structure in our dataset. A comparison of spatial and temporal data of *rbcL_67* lineages identified in modern and past sediments gave additional support for this assumption. Seven (*a67/c67_01* to *a67/c67_07*) lineages were retrieved from both datasets, seven (*a67_08* to *a67_14*) were detected only in modern sediments and five lineages (*c67_15* to *c67_20*) were obtained only from cored sediments. Lineages present in sediment slices with a high *Larix* pollen percentage (>5%, lower part of the core) are mostly identical or similar (only one to two nucleotide differences) to lineages found in modern forested lakes, e.g. *a/c67_07*, *a/c67_04*, *a67_04* and *c67_17*, *a67_03/10* and *c67_20*. Lineages mainly retrieved from modern tundra lakes (*a67_08*, *a67_09*) differed in only one to two nucleotides from *c67_16*, a lineage occurring only at a very low *Larix* pollen percentage in the sediment core (Fig. 3). Moreover, dbRDA results show significant correlations between the *rbcl_c67* lineages’ evolutionary relatedness and *Larix* pollen in the sediment core. This indicates that more closely-related *Staurosira* lineages occur in periods with similar vegetation (Table 2 and Fig. 4).

### Microscopic diatom analyses

Light-microscopic investigations on sediment core 11-CH-12A provided morphological evidence of *Staurosira* species being present in the sediments investigated genetically. The light-microscopic survey noted the presence of small benthic fragilarioids throughout the entire core, but dominant in the upper 40 cm of the core (up to 80% of all diatoms detected, Supplementary Fig. S5). The dominant taxa (making up 33% of all diatom counts) within the fragilarioid group are *Staurosira construens*, *Staurosira venter, Staurosirella pinnata*, and *Pseudostaurosira* sp. (comprising most dominantly *Pseudostaurosira brevistriata* and four rarely detected species *Pseudostaurosira elliptica, P. subsalina*, *P. parasitica, P. pseudoconstruens)*. Other fragilarioid species, namely *Staurosira bidens*, *Pseudostaurosira elliptica*, and *Staurosirella lapponica*, occurred rarely and made up only 0.4% of all recorded diatoms. Within the fragilarioid species, *Staurosirella pinnata* and *Staurosira venter* dominate throughout the core, whereas *Staurosirella pinnata* is predominant in the upper part and *Staurosira venter* is predominant in the lower section.

The turnover of lineages along with vegetation change is also documented by morphologically identified *Staurosira* taxa that change from predominately *Staurosira venter* in the lower part of the core characterized by a high *Larix* pollen percentage to a dominance of *Staurosirella pinnata* in the upper section with a low *Larix* pollen percentage (Supplementary Fig. S5).

These temporal changes were also detected with SEM inspections (Supplementary Fig. S5). Using SEM we selectively identified the following fragilarioid taxa: *Pseudostaurosira* sp., *P. pseudoconstruens*, *Staurosira venter* form 1 and 2 and *Staurosirella pinnata* form 1 and 2 (Supplementary Fig. S6). The two forms of *S. venter* and *S. pinnata* co-occur in most samples, and along with the light-microscopy inspection, we identified a dominance of *S. pinnata* in the upper part, whereas *S. venter* is predominant in the lower section. However, genetic and microscopic assessments are partially different regarding the diversity in fragilarioid taxa. These differences are due to the limitation of genetic reference data, the resolution of the applied genetic marker, and the potential filtering of the applied genetic approach, as the different steps, i.e., DNA isolation, PCR and cloning, may selectively enrich the targeted and most dominant DNA in the investigated samples. Nonetheless, the general turnover in *Staurosira* taxa/lineages over the last 7000 years was detected with both microscopic and genetic methods. Higher diversity (intraspecific variation) and insight into the evolutionary relationships between the different closely-related *Staurosira* lineages were only facilitated by the genetic approach.

## Discussion

Microscopic studies of modern diatom assemblages revealed a strong community turnover across the Siberian treeline ecotone^20^. Environmentally related turnover in *Staurosira* taxa is also displayed throughout Holocene deposits recovered from a Siberian lake^22^. Our genetic study confirms this discovery as we detected a significant correlation between the occurrence of *Staurosira* lineages and vegetation type in modern samples, and temporal variation of *Staurosira* lineages, also supported by microscopic inspections, matching vegetation change identified in core sediments.

Phylogenetic studies applying *rbcL* as a genetic marker have been used to identify diatom taxa and revealed interspecific and intraspecific variants among morphologically similar types originating from different habitats^34^,^35^. The genetic determination of diatom taxa facilitated the detection of biogeographic patterns in diatoms^36^,^37^, which were not detectable with traditional morphological surveys^26^. Our data indicate a high genetic diversity displayed in intra/inter-specific variation in *Staurosira* that correlates strongly with environmental variables and very weakly with geographic patterns (Supplementary Fig. S3, Table S5), but these variables, as found in other diatom studies, cannot be easily separated^3^. Additional investigations on historical patterns can help to disentangle both variables, but often this information is not available^9^,^36^. We achieved this by including historical genetic information from a sediment core (covering the last 7000 years) from a single locality that has experienced profound shifts in ecological conditions, which are similar to those seen in geographical space today. Using ancient sedimentary DNA we directly analysed past diversity in *Staurosira* lineages from different sediment horizons. Some of the ancient lineages obtained from our data are identical to lineages detected in recent sediments, but other unique lineages also occurred, which might indicate differentiation within the sampled lake. In this way, studies on ancient DNA from sedimentary deposits can go beyond classical palaeoecological studies, as they offer the opportunity to track temporal changes of intra/inter-specific lineages (high taxonomic resolution) within aquatic habitats^14^,^15^,^38^, which remain undetectable using microscopic methods.

Beyond unveiling hidden diversity in modern and past diatom communities, our study focussed on the investigation of evolutionary relationships between the *Staurosira* lineages. In modern sediments we detected more closely-related lineages occurring in similar vegetation types and less closely-related lineages present in different vegetation types. In core sediments from a single location, lineages present in similar vegetation (as indicated by *Larix* pollen percentages) were more closely related than those occurring in periods with dissimilar vegetation. Additionally, the comparison of modern and ancient *Staurosira* lineages indicated that lineages that occurred in former forests are closely related to lineages present in contemporary forests. Thus, the combination of modern and historical genetic data provides evidence that environmental conditions, rather than geographic distances are shaping the relatedness patterns of *Staurosira* lineages across the Siberian treeline and through the Holocene time period.

Although our study does not point to where and when the original differentiation of *Staurosira* lineages occurred, we tentatively speculate that the differentiation of *Staurosira* lineages might have occurred in the circum-Arctic, because *Staurosira* shows a very high abundance and diversity in this realm. We speculate that the observed lineages relatedness pattern might be a result of the adaptation to diverse environmental conditions across the arctic-boreal treeline (not necessarily in Siberia), which evolved since the late Pliocene^39^. Furthermore, the observed lineages relatedness pattern might originate from geographically-driven divergence and subsequent repopulation of ecologically different habitats. Such geographically-driven processes are related to recurrent habitat disconnection such as that arsing in the course of past glacial/interglacial climatic cycles^36^,^40^. For example, sea level rise separated the North American and Asian continents during interglacial periods^41^.

To our knowledge, only one study exists that examines strong genetic and phenotypic differentiation across the Siberian treeline, revealing a divergence among tundra and taiga wolf populations caused by prey-habitat specialization^42^. This indicates that the (Siberian) treeline can function as an ecological boundary potentially providing a parapatric (speciation) setting with strikingly different ecological conditions on either side, and potentially promoting divergent adaptation and – ultimately – speciation. These evolutionary processes have been detected along ecological gradients in other locations^43^,^44^, but no genetic study exists about diatoms in treeline ecotones.

In general, our implemented study design provides an opportunity to portray recent and past intra/inter-specific genetic diversity and patterns of evolutionary relationships in minute eukaryotic organisms sensitive to environmental change. Recent global warming propagates the northward extension of the treeline^45^,^46^ and will probably lead to the extension of present and the formation of new water bodies. Such changes might create different environmental gradients and conditions across the treeline and microbes in the embedded waters are putatively affected by these changes^47^. An extension of the applied approach in terms of sampling area and genetic data, i.e. (meta)genomic approaches, could provide information about short-term biodiversity changes and insight into the differentiation of new genetic lineages and speciation in eukaryotic microbes reflecting possible shifts in large-scale biogeographic patterns.

## Methods

### Sampling localities

Sediments and water samples were collected from twenty lakes along three latitudinal transects in the northern lowlands of the federal subjects Yakutia (Sakha Republic) and Krasnoyarsk Krai in Russia (Fig. 1 and Table 1). The localities cover the current treeline ecotone as they range from Arctic tundra near the Laptev Sea coastline, to the forest–tundra and northern taiga forests south–southeast of Taymyr peninsula, including lakes from the Khatanga (CH) region, belonging to Krasnojarsk Krai, and lakes from the Saskylakh (SA) and Tiksi (Tik) regions in Yakutia. The investigated areas are characterized by continuous ice-rich permafrost. The investigated lakes were mostly formed by thermokarst processes and are freshwater, oligotrophic, shallow and small both in size and catchment area. The twenty investigated lakes were assigned to four defined vegetation types: arctic tundra, single-tree tundra, forest-tundra and light northern taiga. These classifications are based on a vegetation map for Yakutia^48^ and field observations. One to three centimetres of surface sediments were taken with a grab sampler. We reduced cross-contamination between sediments by sampling the lakes on different days and washing the sediment grab several times before sampling. The grab content was subsampled and transferred into sterile Whirl-Pak^®^ plastic bags and transported from field to laboratory and stored in the dark at 7°C at the Alfred Wegener Institute. For the genetic assessment, small quantities from each sample were transferred with a sterile spatula into sterile Falcon tubes and stored at -20°C in the historic DNA laboratory at the University of Potsdam for further processing. The sediment core (11-CH-12A) with a length of about 132 cm and 8 cm in diameter was collected with a UWITEC® coring system. The core was taken unopened from field to laboratory (Alfred Wegener Institute), cut into two halves; one half was sampled for pollen and diatom valve analysis and ^14^C dating material and the other half for DNA and geochemical analyses. Diatoms were morphologically analysed on about 0.02 g of cored sediment (Supplementary Methods). The second core half was sampled each centimetre and samples were taken under a fume hood and with a sterile spatula, cleaned thoroughly with DNA-Exitus Plus^TM^ and Ethanol before and in-between each sampling. For DNA sampling the inner part of a sediment slice was again sampled with a cleaned spatula, transferred to sterile tubes and mixed thoroughly with Queens Tissue Buffer^49^ and stored in the dark at 10°C. Sampling was carried out in 2007, 2009 and 2011, during field campaigns conducted by the Alfred Wegener Institute, Potsdam, in cooperation with the North-Eastern Federal University of Yakutsk.

### Sediment core chronology

In total, sixteen samples of the 11-CH12-A core were radiocarbon dated (Supplementary Table S1) and thirteen of these dates were used to establish an age-depth model using the Bacon package^50^ in the R software, where the calibrated ages are based on IntCal13^51^. According to the established age-depth model the analysed core dates go back approximately to the mid Holocene around 7000 Yr. BP (Supplementary Fig. S1). The calculated average sedimentation rate of 0.025 cm/Yr. fits well to the recent sedimentation rate inferred from the ^210^Pb/^137^Cs dating (performed at University of Liverpool) of a parallel short core of 0.03 cm/Yr. for the last 180 years (Supplementary Fig. S1).

### Pollen analysis

Relative *Larix* pollen frequency was obtained from results of pollen analyses that were performed on 65 fossil sediment samples. Samples of 1.5 ml underwent standard procedures including treatments with HCL, KOH, HF, acetolysis, and mounting in glycerine. At least 500 terrestrial pollen grains were counted in each sample at ×400 magnification.

### Genetic assessment

All DNA isolations were conducted under a DNA-Isolation-UV-Hood placed in a dedicated historic DNA laboratory at the University of Potsdam, physically separated from the general genetic laboratories to prevent contamination by DNA from modern samples and PCR amplifications. Core sediment samples that were extracted for DNA are given in Fig. 4. PCRs were set up under a Pre-PCR-UV-Hood placed in the historic DNA laboratory at the University of Potsdam. Amplicons, *rbcL_194* and *rbcL_191*, were produced using newly designed taxon-specific primers for the amplification of fragilarioid DNA from surface sediments. Shorter amplicons, *rbcL_76* and *rbcL_67*, were targeted by group-specific primers that amplify DNA from various diatom species^15^ and were used for core sediment amplifications. All primer pairs were analysed with regard to their taxonomic specificity using an *in silico* PCR approach^52^ (Supplementary Table S2). All positive PCR products were cloned, and as many positive clones as available were sequenced. For full details see Supplementary Methods.

We applied a stringent sequence verification protocol to exclude sequences derived from polymerase errors. Initial taxonomic assignment was performed using BLAST and phylogenetic relationships using Bayesian inference. Haplotype networks and uncorrected nucleotide distances were computed for sequences taxonomically belonging to *Staurosira* relatives only. These sequences were named *Staurosira* lineages. For full details see Supplementary Methods. DNA sequences of Staurosira lineages retrieved from sediment samples are deposited in PANGAEA (http://doi.pangaea.de/10.1594/PANGAEA.848399).

### Ordination analysis

We performed non-metric multidimensional scaling (NMDS; with a Bray-Curtis distance measure) to portray the major patterns of the occurrence of *Staurosira* lineages (i.e., presence/absence data for *rbcL_191* and *rbcL_a67*). Furthermore, we conducted redundancy analyses (RDA) to investigate the correlation between the lineages’ occurrences and geographic distance (using dummy variables for coding the lakes’ location in one of the three north-south transects) and vegetation (using dummy variables for coding the lake’s location in one of the four vegetation types). We describe the biogeographic preference of each *Staurosira* lineage by calculating the transect affinity of each *Staurosira* lineage, which is the mean relative frequency of each lineage in the three transects, corrected for the number of lakes belonging to each transect. We describe the vegetation preference of each *Staurosira* lineage by calculating the vegetation type affinity, which is the mean relative frequency of each lineage in the four vegetation types, corrected for the number of lakes belonging to each vegetation type. Using distance-based RDA (dbRDA,) we then calculated the correlations between the lineages’ patristic distances (=pairwise distances between each pair of sequence type, which are based on the tree branch lengths extracted from the Bayesian inference) and the lineages’ transect affinity (=pairwise distances of lineages’ transect affinity) and the lineages’ vegetation type affinity (=pairwise distances of lineages’ vegetation type). These tests were used to infer whether more closely-related lineages occur preferentially within the same transect or vegetation types than distant lineages. A correlation between the lineages’ patristic distances from the sediment core and past vegetation (defined by the *Larix* pollen affinity, i.e. the mean relative *Larix* pollen percentage at which each *rbcL_c67* sequence type occurred) was performed to infer whether more closely related *rbcL_c67* sequences occurred along with similar past vegetation condition. All matrices used in the analyses were based on presence/absence data of the obtained *Staurosira* lineages. For RDA and dbRDA we applied forward selection (p < 0.1) to identify the final dummy variable set in each model. We neglected the relative frequencies of lineages because they are only semi-quantitative as they may be strongly biased by PCR effects^53^ and depend on the number of clones investigated. NMDS, RDA and dbRDA analyses were performed using R vegan package 1.17.

## Additional Information

**How to cite this article**: Stoof-Leichsenring, K. R. *et al*. Genetic data from algae sedimentary DNA reflect the influence of environment over geography. *Sci. Rep*. **5**, 12924; doi: 10.1038/srep12924 (2015).

## Supplementary Material

Supplementary Information

## Figures and Tables

**Table 1:** Physical and hydrochemical characteristics and vegetation-based parameters (leaf area index (LAI), *Larix* sp. pollen percentage, vegetation type (arctic tundra; single-tree tundra; forest tundra; light northern taiga) of the investigated lakes.

Lakes	Latitude (°E)	Longitude (°N)	Size (ha)	Depth (m)	Secchi depth (m)	pH	Conductivity (μS/cm)	HCO_3_^−^ (mg/l)	LAI median	*Larix* pollen (%)	Vegetation type
09-Tik-05	71.294	125.550	0.160	5.0	2.5	7.00	67.0	34.3	1.260	1.75	arctic tundra
09-Tik-08	72.245	125.630	0.480	2.9	1.0	6.70	32.0	15.7	0.796	0.54	arctic tundra
09-Tik-09	70.698	125.074	0.640	6.3	1.6	6.20	14.0	5.0	1.238	2.88	light northern taiga
09-Tik-13	69.405	123.828	0.640	2.5	1.0	7.23	27.0	17.8	1.412	4.47	light northern taiga
09-Tik-14	70.925	125.557	0.240	6.3	1.5	6.57	27.0	12.8	1.304	3.53	light northern taiga
07-SA-26	72.320	111.189	0.160	6.8	4.5	7.32	30.0	16.2	1.118	2.62	arctic tundra
07-SA-31	72.071	111.118	0.023	6.9	1.8	7.15	20.0	7.0	1.122	4.41	single-tree tundra
07-SA-33	71.747	111.132	0.240	5.0	1.5	7.20	38.0	19.2	1.182	3.77	light northern taiga
11-CH-02	71.836	102.883	0.060	3.5	1.7	6.97	51.1	33.3	1.180	2.02	forest tundra
11-CH-06	70.667	97.716	0.045	4.8	2.5	6.42	35.3	23.0	1.510	1.94	light northern taiga
11-CH-09	70.670	97.716	0.020	4.8	3.0	5.09	43.2	24.7	1.510	2.97	light northern taiga
11-CH-10	70.673	97.726	0.105	15.4	4.0	7.09	40.7	23.2	1.512	3.06	light northern taiga
11-CH-11	70.901	97.649	0.030	19.7	4.7	6.24	47.2	30.1	1.432	1.29	single-tree tundra
11-CH-12	72.399	102.289	0.030	14.3	5.0	7.50	34.9	31.3	1.060	1.65	single-tree tundra
11-CH-13	72.380	102.281	0.012	11.1	3.7	6.31	80.1	49.9	1.058	1.65	single-tree tundra
11-CH-14	72.398	102.288	0.005	6.7	2.5	7.38	39.9	27.5	1.060	1.71	single-tree tundra
11-CH-15	72.403	102.261	0.045	4.1	2.0	6.90	45.6	23.6	1.060	2.97	single-tree tundra
11-CH-17	72.245	102.236	0.022	3.4	1.7	7.87	64.2	25.0	1.060	1.74	forest tundra
11-CH-18	72.307	102.375	0.045	4.8	1.3	8.02	59.0	32.0	1.062	5.42	forest tundra
11-CH-19	72.255	102.213	0.053	4.4	1.9	7.84	183.2	111.7	1.066	3.87	forest tundra
11-CH-20	72.258	102.216	0.045	2.7	1.7	7.92	68.3	41.8	1.064	2.58	forest tundra
Median			0.045	5.0	1.9	7.09	40.7	24.7	1.122	2.62	
1st quartile			0.030	4.1	1.6	6.57	32.0	17.8	1.060	1.74	
3rd quartile			0.160	6.8	3.0	7.38	59.0	32.0	1.304	3.53	

**Table 2:** Distance-based redundancy analyses results.

Data	Explanatory set	Forward selection	R^2^	adR^2^	*P*
Testing the correlation between lineages’ phylogenetic distance vs. lineages’ transect affinity or lineages’ vegetation type affinity					
*rbcl_67* phylog. distance	Lineages’ transect affinity	CH affinity	18.4%	11.6%	0.053
*rbcl_67* phylog. distance	Lineages’ vegetation type affinity	Tundra & single tree tundra affinity	40.3%	29.4%	0.01*
*rbcL _191* phylog. distance	Lineages’ transect affinity	SA affinity	7.3%	2.8%	0.196
*rbcL _191* phylog. distance	Lineages’ vegetation type affinity	Single tree tundra affinity	14.6%	10.5%	0.014*
Testing the correlation between lineages’ nucleotide sequences vs. lineages’ *Larix* pollen affinity					
*rbcL _c67* phylog. distance	Lineages’ *Larix* pollen affinity	*Larix* pollen affinity	22.2%	15.2%	0.001*

^*^Statistically significant *P* values (*P* < 0.05).
