# Supplementary material for: Genetic data from algae sedimentary DNA reflect the influence of environment over geography
Source: Sci Rep. 2015 Aug 11;5:12924. doi: 10.1038/srep12924 (PMC4542542; doi:10.1038/srep12924)
Supplement: Supplementary Information [file srep12924-s1.doc]

Supplementary Information

Title:

Genetic data from algae sedimentary DNA reflect the influence of environment over geography

Kathleen R. Stoof-Leichsenring^1^, Ulrike Herzschuh^1,4*^, Luidmila A. Pestryakova^3^, Juliane Klemm^1^, Laura S. Epp^1^, Ralph Tiedemann^2*^

Supplementary Methods

**Diatom analysis**. Diatoms were morphologically analyzed on about 0.02 g of cored sediment. Calcareous and organic components were removed by heating with HCl (10%) and H_2_O_2_ (30%), respectively. Purified diatom samples were mounted on microscope slides using Naphrax®. Valves were counted along a defined transect using a Zeiss microscope at a magnification of ×1000. The literature used for diatom determination is listed in [^1^](#_ENREF_1). Inspections using the scanning electron microscope (SEM, Zeiss Gemini**®** Ultra plus), with 7.3 mm working distance and 10.00 kV, were performed solely on small fragilarioid diatoms from selected core sediment samples, therefore diatom preparations were subsampled, coated with a gold/palladium alloy (Au/Pd) and analyzed at GFZ Potsdam.

**Genetic assessment.** All DNA isolations were conducted under a DNA-Isolation-UV-Hood placed in a dedicated historic DNA laboratory at the University of Potsdam, separate from the general genetic laboratories preventing contamination by DNA from modern samples and PCR amplifications. The UV-Hood was cleaned with Ethanol and DNA-Exitus Plus^TM^ and 30 min UV sterilization before and after use. All required instruments including pipettes, pipette tips and reaction tubes were placed under the hood for UV sterilization. Isolation of surface sediment samples was performed using PowerSoil Max™ DNA Isolation Kit (MoBio Laboratories, California). Between 6 and 8 g of wet sediment was transferred to the PowerSoil™ bead solution tubes and was shaken together with 1.2 ml of C1 buffer and 0.8 mg Proteinase K in a water bath over night at 65°C. The next day, the solution was centrifuged at 2500 g for 3 min, the supernatant was discarded and samples were mixed with 5 ml of C2 buffer. The following steps were performed according to the manual’s instructions. DNA was eluted in 2x800 μl of C6 buffer and isolates were stored in 200 μl aliquots at -20°C until required. DNA Isolations from different localities (CH, SA, Tik) were extracted on different days to prevent cross-contamination. A maximum of seven sediment samples and one blank control were isolated within one round. DNA from cored sediment samples was extracted using PowerSoil™ DNA Isolation Kit (MoBio Laboratories, California). The PowerSoil™ bead solution was transferred to a sterile 2.0 ml reaction tube. The samples with QTB were thoroughly mixed and 500 μl of each sample-buffer mixture was transferred to the bead solution tube. Each sample was centrifuged at 10,000 g for 1 minute and the supernatant was removed, leaving 150–200 mg of sediment. The PowerSoil™ bead solution was re-transferred to the sediment tube, 60 μl C1 buffer was added and the solution shaken for 3 minutes at 1000/min using a Mini-BeadBeater. The following steps were conducted according the manual’s instructions. DNA was eluted in 50 μl C6 buffer. Isolated DNA was stored at ‑20°C. A maximum of nine core sediment samples and one blank control were extracted in one round. To ensure the authenticity of results when working with ancient or degraded environmental DNA we included blank controls for isolations, PCR and nested PCR.

**Primer specificity.** All primer pairs were analyzed according to their taxonomic specificity using *in silico* PCR approach [^2^](#_ENREF_2). For the *in silico* PCR we used a database compiled from the entire EMBL Nucleotide Sequence Database, downloaded from ftp://ftp.ebi.ac.uk/pub /databases/embl/release/ (release embl_117, September 2013) and the tested primers. The output file of the program lists all sequences that could potentially be amplified by the tested primers and provides taxonomic information about the sequence entries. For the *in silico* PCR we applied the following parameters: (1) amplicon lengths of between 20 and 1,000 base pairs, and (2) a maximum of two errors between the query sequence and the primer sequence, but no errors allowed in the last two bases at the 3’ end. We estimated the specificity of the primers to diatoms by calculating the percentage of diatoms within the *in silico* amplicons, excluding sequences with uncertain taxonomic determination.

**PCRs** were set up under a Pre-PCR-UV-Hood placed in the historic DNA laboratory at the University of Potsdam. PCR reactions for surface and cored sediments were run in a two-step approach (nested PCR). For surface sediments 194 (*rbcL_194*) and 191 (*rbcL_191*) base pair (bp) long fragments of the *rbcL*, and for core sediments shorter 76 and 67 bp fragments of the *rbcL*, were amplified. Amplicons, *rbcL_194* and *rbcL_191*, were conducted using newly designed species-specific primers for the amplification of fragilarioid DNA, especially from *Straurosira* taxa. For primer design we used a longer *rbcL* fragment of *Straurosira* and *Staurosirella* lineages obtained from Siberian lakes sediments in an earlier study [^3^](#_ENREF_3) and modified the primers according to specific nucleotide substitutions. Shorter amplicons, *rbcL_76* and *rbcL_67*, were targeted by group-specific primers that amplify DNA from various diatom species [^4^](#_ENREF_4) (Supplementary Table S2). All PCR reactions were run in the PCR area of the general genetic laboratory. Amplifications of core samples and surface samples were run on different days and in different thermo cyclers. PCRs on core sediment extractions were run in a dedicated TPersonal thermal cycler (Biometra, Göttingen, Germany), used only for ancient DNA amplifications, placed under a separate UV-Hood, which was cleaned with DNA-Exitus Plus^TM^ and UV light sterilization between different PCR runs. For surface sediment PCR runs we used a T3000 thermal cycler (Biometra, Göttingen, Germany). The following reagents were used for each amplification: fragment-specific primers (each at a final concentration of 0.25 mM), 10 x PCR buffer containing MgCl_2_, 0.5 mM of each dNTP and 0.5 U *Taq* polymerase (Taq DNA CORE Kit 10, MP Biomedicals). For the first (initial) PCR we added 3 μl of DNA template solution to the reaction mix, whereas 1 μl of PCR product from the first PCR was added to the second (nested) PCR as a template. All amplifications were performed according to the following reaction profile: reactions were initially heated to 94°C, followed by one cycle at 94°C for 5 minutes, and then 35 cycles each at 94°C for 30 seconds, at 58°C (*rbcL_194*, *rbcL_191*) and 43.6°C (*rbcL_76*, *rbcL_67*) for 30 seconds, at 72°C for 30 seconds, and a final extension at 72°C for 10 minutes.

**Cloning.** All positive PCR products were cloned using the TOPO® TA Cloning® Kit for sequencing (Invitrogen, Carlsbad, CA, USA). As many positive clones as available (up to a maximum of 48 per sediment sample) were sequenced.

**Sequence verification, taxonomic identification and phylogenetic inferences.** *RbcL* sequences retrieved from cloning were aligned using Clustal W with default settings as implemented in BioEdit version 7.2.0. [^5^](#_ENREF_5). Sequence fragments of vector DNA and primers were excised manually. Initial taxonomic assignment was based on a simple nucleotide BLAST® (Basic Local Alignment Search Tool) search using the *Blastn* or *megablast* algorithm. Subsequently, within our dataset, subsets of sequences taxonomically assigned to *Staurosira* were extracted and analyzed further. Sequences with different taxonomic assignments were excluded from these subsequent analyses. *Staurosira* sequences obtained from surface and core samples were defined as authentic if they occurred in two independent reactions and more than once in one of the reactions. Rare sequences obtained from *rbcL_191* amplicons (= sequences present in more than one clone, but found only in a single reaction) were considered authentic if they showed more than one polymorphism at polymorphic sites in comparison to other obtained and referenced sequences in GenBank. Rare sequences obtained from *rbcL_67* amplicons (= sequences occurring only once in two independent reactions) were considered authentic if they showed at least one nucleotide polymorphism at a polymorphic site in comparison to other obtained and referenced sequences in GenBank. Rare and single sequences that showed unique nucleotide substitutions in otherwise non-polymorphic positions were considered to have resulted from polymerase errors and were excluded from the dataset. After sequence evaluation, the verified *Staurosira* sequences were named *Staurosira* lineages. To facilitate a comparison of surface and core datasets, verified *rbcL_191* lineages from surface sediments were shortened to the length of the *rbcL_67* amplicon. The shorter (67 bp) lineages derived from *rbcL_191* lineages were named *rbcL_a67*, whereas the lineages retrieved from core sediments were called *rbcL_c67*. Prior to the phylogenetic analyses, we estimated the HKY+G model as best fit model for the underlying sequence datasets (*rbcL_191* and *rbcL_a67/c67* including reference sequences used in phylogenetic inferences) using jMODELTEST 2.1.4 [^6^](#_ENREF_6). With the program we evaluated 88 models of evolution and used the AIC, implemented in jMODELTEST 2.1.4 to perform a statistical selection among the optimized models. Bayesian phylogenetic analyses were conducted for the *rbcL_191*, and the combined data of *rbcL_a67* and *rbcL_c67* lineages, with both trees including eleven related lineages from araphid diatoms and *Cyclotella* *choctawhatcheeana* (JQ217351) as the outgroup. We calculated phylogenetic trees using MrBayes (Huelsenbeck) applying the estimated model of evolution and performed two independent runs for 2,000,000 generations. Both sets of *rbcL* lineages were then analyzed with TCS software to reconstruct 95% probability parsimony networks inferring genealogies at levels of low divergence [^7^](#_ENREF_7). The uncorrected pairwise nucleotide distances between each pair of *rbcL_191*, *rbcL_a67* and *rbcL_c67* lineages were calculated in MEGA 5.2 [^8^](#_ENREF_8). Uncorrected pairwise nucleotide distances were used to infer the evolutionary relatedness between the *Staurosira* lineages.

**Table S1**. Radiocarbon dates for the sediment core 11-CH-12A.

Dates from 5.5, 98.5 and 101.5 cm were considered outliers and

were not used to construct the age-depth model.

| **Mean sample depth (cm)** | **Dated material** | **Weight of material (g)** | **^14^C age (BP)** |
| --- | --- | --- | --- |
| 5.5 | bulk | 0.3098 | 1280±40 |
| 26.5 | moss | 0.0360 | 870±40 |
| 35.5 | moss | 0.0150 | 1645±35 |
| 50.5 | bulk | 0.2710 | 2105±30 |
| 60.5 | moss | 0.0025 | 2375±35 |
| 65.5 | bulk | 0.7550 | 3190±35 |
| 71.5 | bulk | 1.2099 | 5075±35 |
| 75.5 | bulk | 0.2474 | 4160±35 |
| 85.5 | bulk | 0.3565 | 5000±50 |
| 98.5 | bulk | 5.0890 | 18240±100 |
| 101.5 | terrestrial material | 0.0080 | 7630±40 |
| 115.5 | wood | 0.0095 | 5750±40 |
| 116.5 | wood | 0.0260 | 5150±50 |
| 119.5 | wood | 0.0064 | 5770±50 |
| 123.5 | bulk | 0.8326 | 5890±50 |
| 131.5 | moss | 0.0480 | 6220±40 |

**Table S2. Primer characteristics and specificity to diatoms.**

| **Primer name and sequence (5′– 3′)** | **Amplicon**  **name** | **Size (bp) (without primer sequence)** | **Primer specificity to diatoms (%)^*^** | **Reference** |
| --- | --- | --- | --- | --- |
| Diat_rbcL_705F  AACAGGTGAAGTTAAAGGTTCATAYTT  Diat_rbcL_808R  TGTAACCCATAACTAAATCGATCAT | *rbcL_76* | 76 | 89.3 | Stoof-Leichsenring et al. (2012) |
| Diat_rbcL_708F  AGGTGAAGTTAAAGGTTCATACTTDAA  Diat_rbcL_802R  CCCATAACTAAATCGATCATAAYRAT | *rbcL_67* | 67 | 69.8 | Stoof-Leichsenring et al. (2012) |
| Sta_rbcl_705F  TACAGGTGAAGTTAAAGGTTCATACTT  Sta_rbcL_927R  GATAACACGGAARTTAATACCATGG | *rbcL_194* | 194 | 90.5 | This study |
| Sta_rbcl_708F  AGGTGAAGTTAAAGGTTCATACTTAAA  Sta_rbcL_926R  ATAACACGGAARTTAATACCATGGT | *rbcL_191* | 191 | 50.9 | This study |

Percentage of diatom (Bacillariophyta) sequences among all *in silico* amplified sequences.

**Table S3.** List of the identified *191_01–23* lineages and

their equivalents after shortening to the length of the

*rbcL_67* fragment; short fragments derived from the

*rbcL_191* lineages are named *a67_01–14*.

| ***rbcL_191*** | ***rbcL_67*** |
| --- | --- |
| *191_01* | *a67_01* |
| *191_02*, *191_12* | *a67_02** |
| *191_07*, *191_11* | *a67_03** |
| *191_13*, *191_05*, *191_06* | *a67_04** |
| *191_21*, *191_19*, *191_08* | *a67_05** |
| *191_20*,*191_22*, *191_15* | *a67_06** |
| *191_03* | *a67_07* |
| *191_17* | *a67_08* |
| *191_04*, *191_23* | *a67_09** |
| *s191_18* | *a67_10* |
| *s191_09* | *a67_11* |
| *s191_10* | *a67_12* |
| *s191_14* | *a67_13* |
| *s191_16* | *a67_14* |

* *RbcL_67* lineages that have more than one equivalent in *rbcL_191*

lineages.

**Table S4.** Taxonomic identification of *191_01–23*, *a67_01–14* and

*c67_01* to *07* and *c67_15* to *20* lineages using BLAST search.

| **Lineages** | **Taxa**  **name** | **Accession**  **number** | **Sequence**  **identity (%)** |
| --- | --- | --- | --- |
| *191_01* | *Staurosira elliptica* | HQ828193.2 | 97 |
| *191_02* | *Staurosira elliptica* | HQ828193.2 | 97 |
| *191_03* | *Staurosira elliptica* | HQ828193.2 | 99 |
| *191_04* | *Staurosira elliptica* | HQ828193.2 | 97 |
| *191_05* | *Staurosira elliptica* | HQ828193.2 | 96 |
| *191_06* | *Staurosira elliptica* | HQ828193.2 | 97 |
| *191_07* | *Staurosira elliptica* | HQ912451.1 | 98 |
| *191_08* | *Staurosira elliptica* | HQ828193.2 | 96 |
| *191_09* | *Staurosira construens* | HQ912451.1 | 99 |
| *191_10* | *Staurosira construens* | HQ912451.1 | 100 |
| *191_11* | *Punctastriata* sp. | HQ828199.2 | 96 |
| *191_12* | *Staurosira elliptica* | HQ828193.2 | 97 |
| *191_13* | *Staurosira elliptica* | HQ828193.2 | 96 |
| *191_14* | *Staurosira elliptica* | HQ828193.2 | 97 |
| *191_15* | *Staurosira construens* | HQ912451.1 | 99 |
| *191_16* | *Staurosira elliptica* | HQ828193.2 | 94 |
| *191_17* | *Punctastriata* sp. | HQ828199.2 | 97 |
| *191_18* | *Punctastriata* sp. | HQ828199.2 | 96 |
| *191_19* | *Staurosira elliptica* | HQ828193.2 | 96 |
| *191_20* | *Staurosira construens* | HQ912451.1 | 99 |
| *191_21* | *Staurosira elliptica* | HQ828193.2 | 95 |
| *191_22* | *Staurosira construens* | HQ912451.1 | 97 |
| *191_23* | *Staurosira elliptica* | HQ828193.2 | 97 |
| *a67_01* ^a^ | *Staurosira elliptica* | HQ828193.2 | 100 |
| *a67_02* ^a^ | *Staurosira elliptica* | HQ828193.2 | 100 |
| *a67_03* ^a^ | *Staurosira elliptica* | HQ828193.2 | 99 |
| *a67_04* ^a^ | *Staurosira elliptica* | HQ828193.2 | 98 |
| *a67_05* ^a^ | *Staurosira elliptica* | HQ828193.2 | 98 |
| *a67_06* ^a^ | *Staurosira construens* | HQ912451.1 | 100 |
| *a67_07* ^a^ | *Staurosira elliptica* | HQ828193.2 | 99 |
| *a67_08* | *Staurosira construens* | HQ912451.1 | 98 |
| *a67_09* | *Staurosira construens* | HQ912451.1 | 97 |
| *a67_10* | *Staurosira elliptica* | HQ828193.2 | 97 |
| *a67_11* | *Staurosira construens* | HQ912451.1 | 99 |
| *a67_12* | *Staurosira construens* | HQ912451.1 | 100 |
| *a67_13* | *Staurosira elliptica* | HQ828193.2 | 97 |
| *a67_14* | *Staurosira elliptica* | HQ828193.2 | 94 |
| *c67_01* ^a^ | *Staurosira elliptica* | HQ828193.2 | 100 |
| *c67_02* ^a^ | *Staurosira elliptica* | HQ828193.2 | 100 |
| *c67_03* ^a^ | *Staurosira elliptica* | HQ828193.2 | 99 |
| *c67_04* ^a^ | *Staurosira elliptica* | HQ828193.2 | 98 |
| *c67_05* ^a^ | *Staurosira elliptica* | HQ828193.2 | 98 |
| *c67_06* ^a^ | *Staurosira construens* | HQ912451.1 | 100 |
| *c67_07* ^a^ | *Staurosira elliptica* | HQ828193.2 | 99 |
| *c67_15* | *Staurosira construens* | HQ912451.1 | 97 |
| *c67_16* | *Staurosira elliptica* | HQ828193.2 | 96 |
| *c67_17* | *Staurosira elliptica* | HQ828193.2 | 97 |
| *c67_18* | *Staurosira construens* | HQ912451.1 | 96 |
| *c67_19* | *Staurosira construens* | HQ912451.1 | 96 |
| *c67_20* | *Staurosira elliptica* | HQ828193.2 | 97 |

**Table S5.** RDA results.

| **Data** | **Explanatory set** | **Forward selection** | **R^2^** | **ad R^2^** | ***P*** |
| --- | --- | --- | --- | --- | --- |
| Testing the correlation between lineages’ occurrences vs. transect or vegetation | | | | | |
| *rbcl_a67* occurrences | transect | SA | 7.7% | 2.5 % | 0.151 |
| *rbcl_a67* occurrences | vegetation | forest + forest tundra | 23.8 % | 14.8 % | 0.004* |
| *rbcL _191* occurrences | transect | SA | 6.7 % | 1.6 % | 0.185 |
| *rbcL _191*  occurrences | vegetation | forest + forest tundra | 22.6 % | 13.4 % | 0.001* |
| Testing the correlation between lineages’ occurrences vs. *Larix* pollen | | | | | |
| *rbcL _c67* occurrences | *Larix* pollen | *Larix* pollen | 5.9% | 0.7% | 0.310* |

***** Statistically significant*P* values (*P* < 0.05)


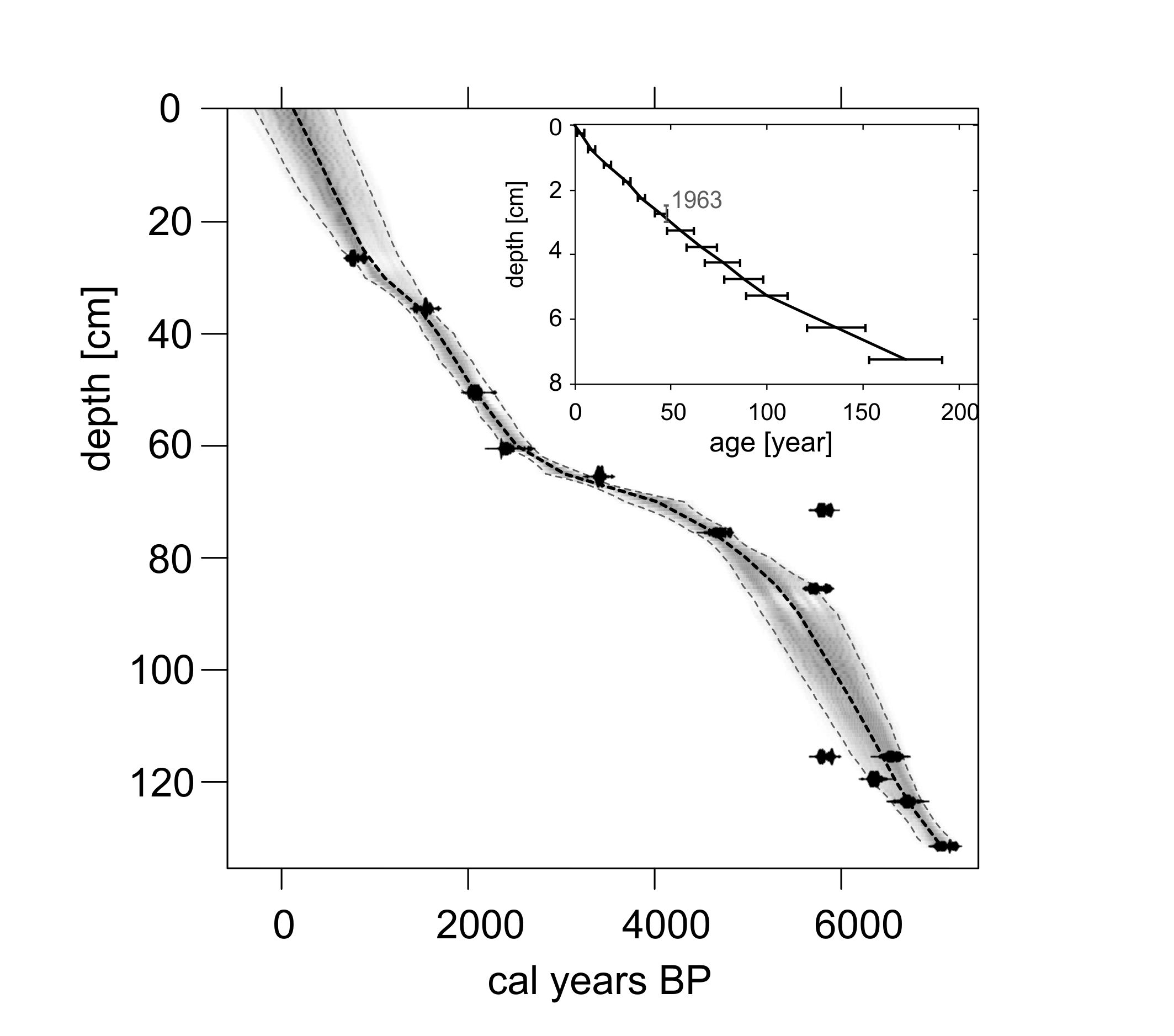


**Fig. S1.**

Age-depth model for the sediment core 11-CH-12A based on thirteen radiocarbon dates (black). Inset panel shows the ^210^Pb/^137^Cs chronology of a parallel short core.

**
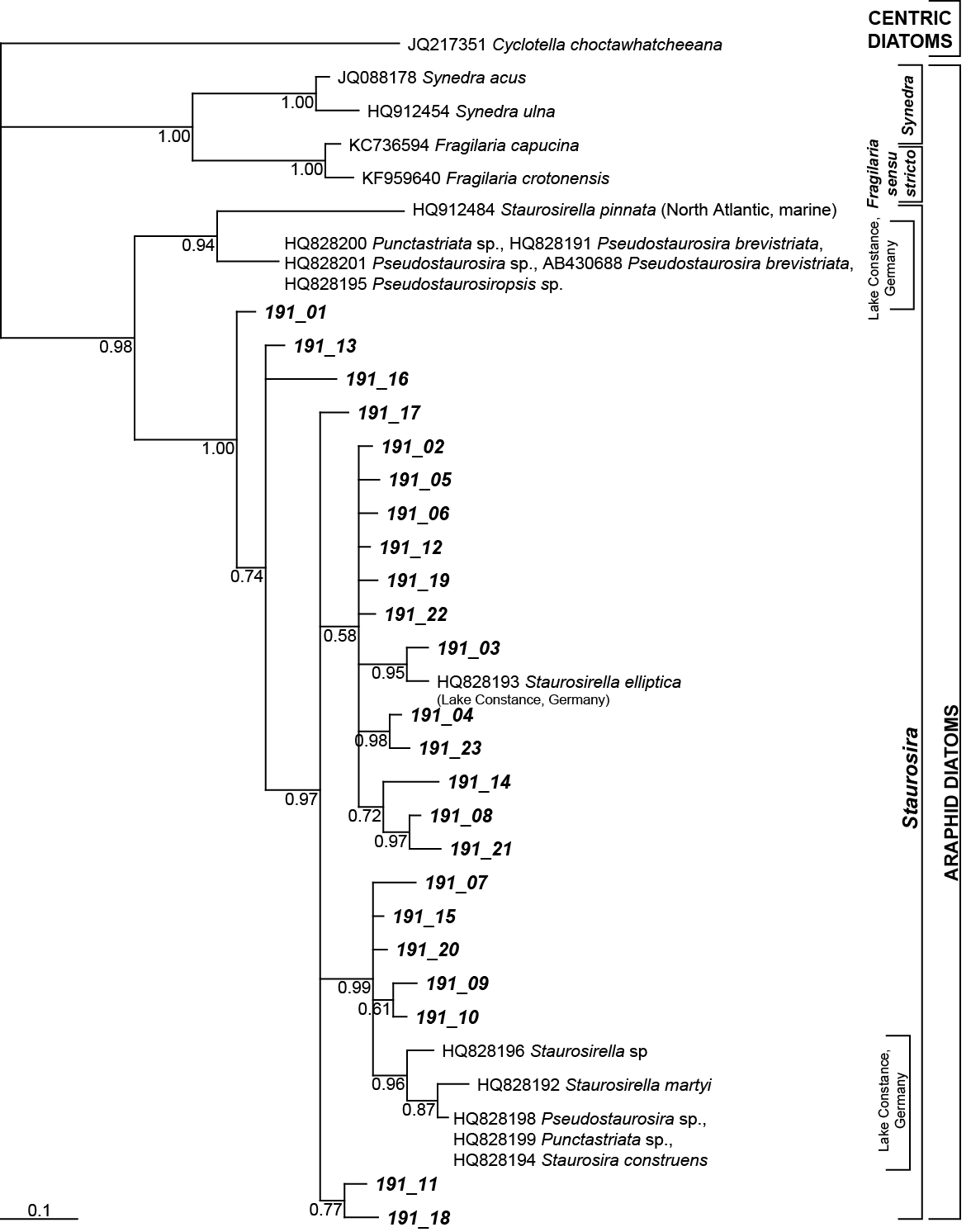
**

**Fig. S2.**

A) Phylogenetic tree (Bayesian analysis, HKY+G model) based on *191_01–23* sequences obtained from Siberian lakes and 13 additional representatives of the Fragilariaceae family and a centric diatom *Cycotella choctawhatcheeana* as outgroup, both obtained from GenBank.


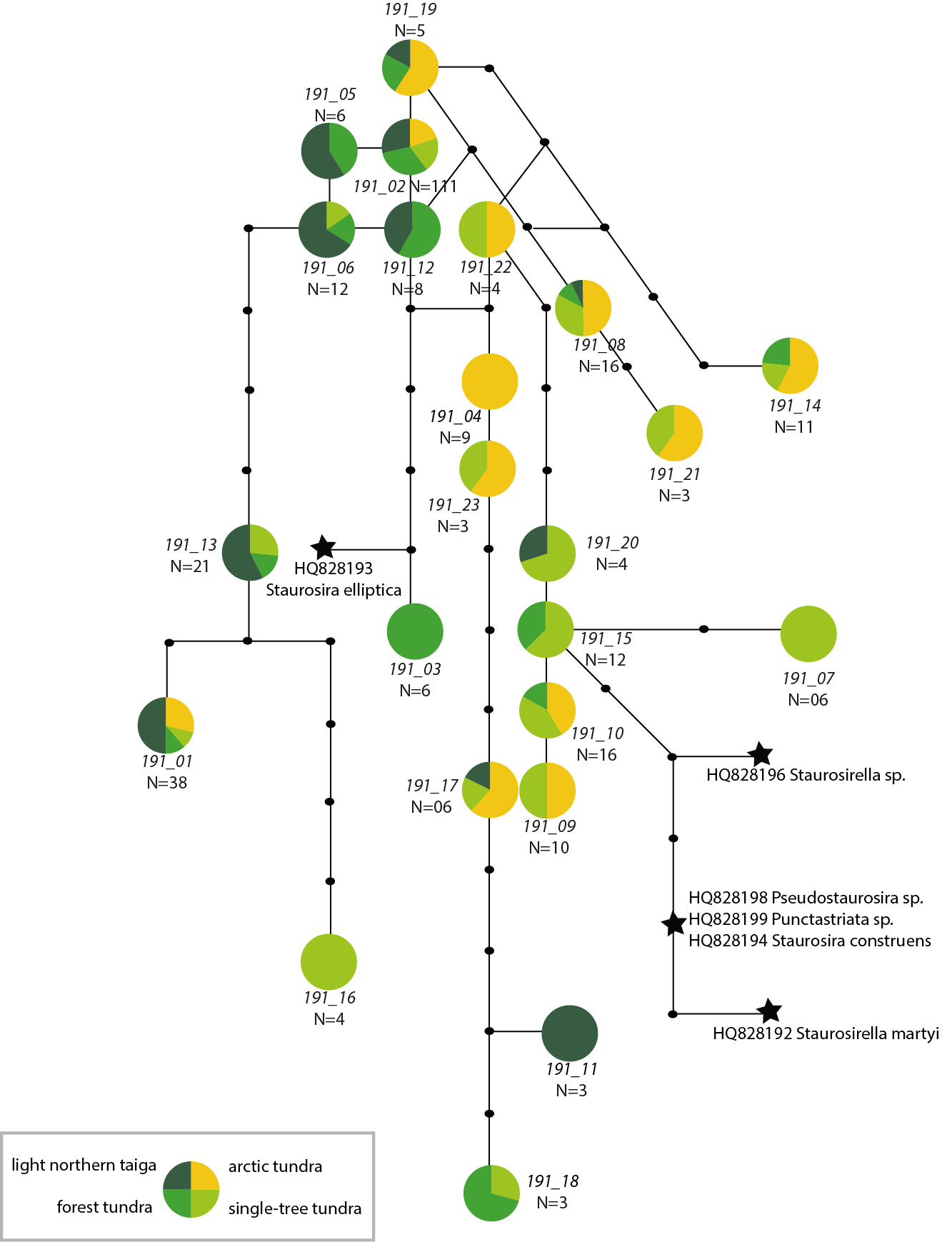


B) Haplotype network containing *191_01–23* lineages and six GenBank entries indicated by a star: HQ828192 *Staurosirella martyi*, HQ828193 *Staurosira elliptica*, HQ828196 *Staurosirella* sp., HQ828198 *Pseudostaurosira* sp., HQ828199 *Punctastriata* sp., HQ828194 *Staurosira construens*. Colored symbols show the proportion of each haplotype in the four vegetation types (see legend). Dots indicate missing haplotypes.


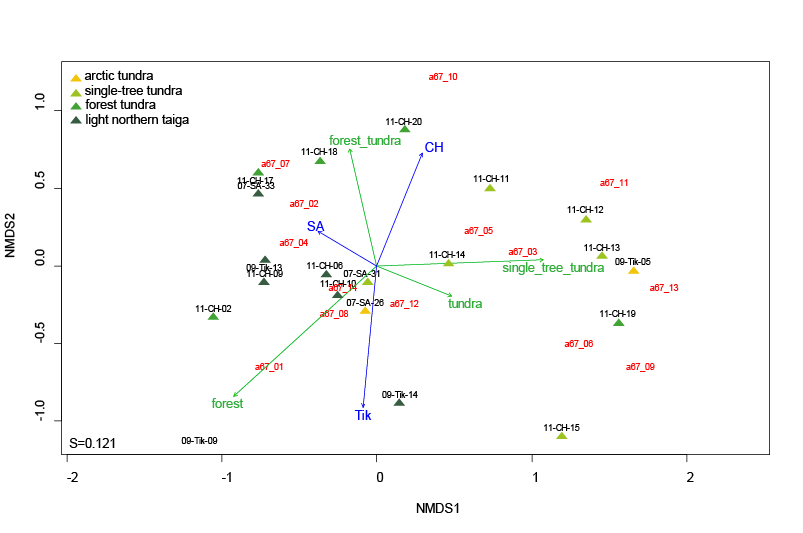


A

B


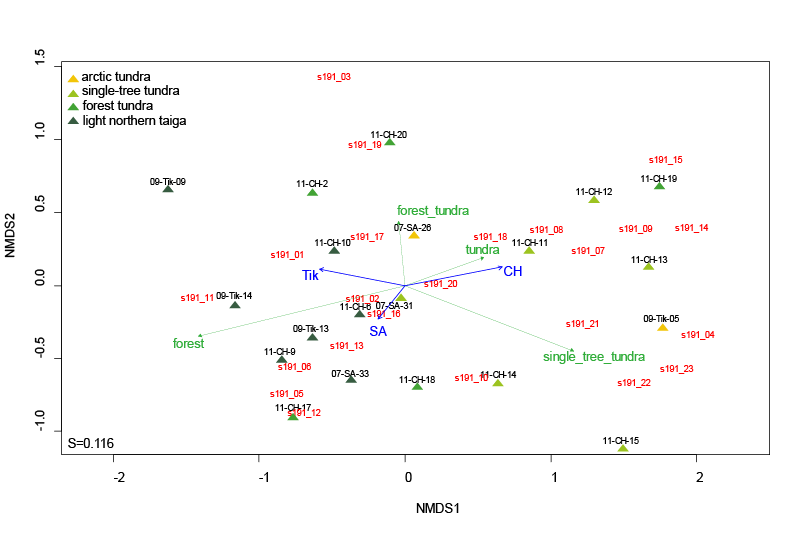


**Fig. S3**

NMDS plots for (A) *rbcL_a67* sequences 01–14 and (B) *191* lineages 01 to 23 (red) alongside lakes (black) and environmental variables (vegetation types (green)=tundra, single-tree tundra, forest tundra, forest; geographic distance (three transects, in blue)=CH, SA and Tik); Colored triangles indicate the vegetation type around each lake. RDA results showed a significant correlation between lineages’ occurrence and vegetation, but not for geographic distance for the two sets of lineages respectively (see Table S5).


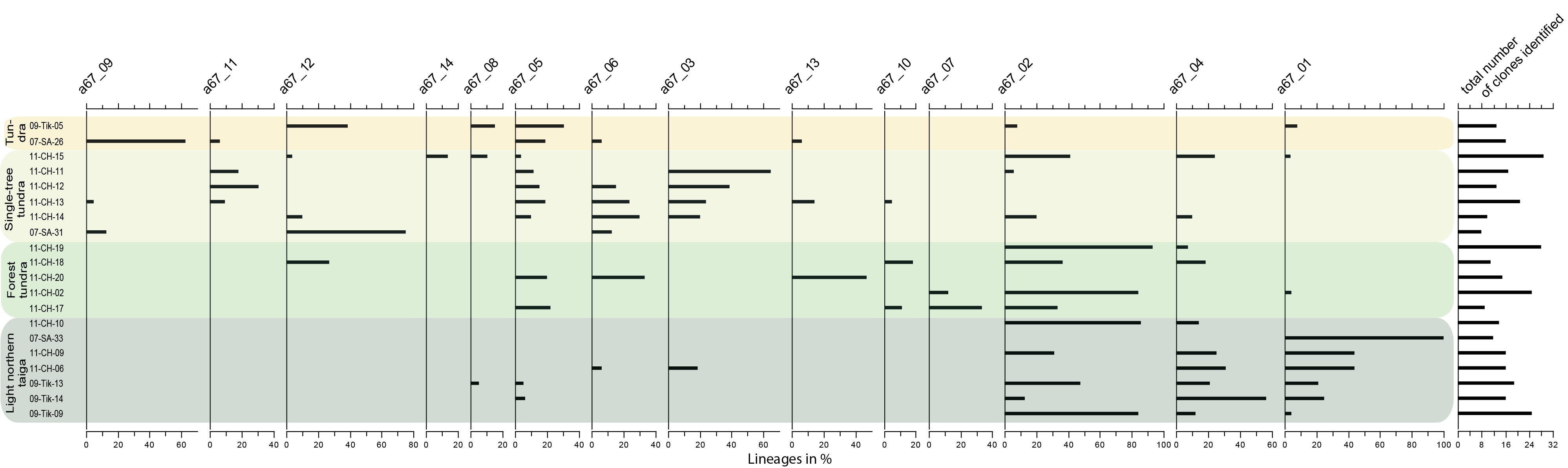


A


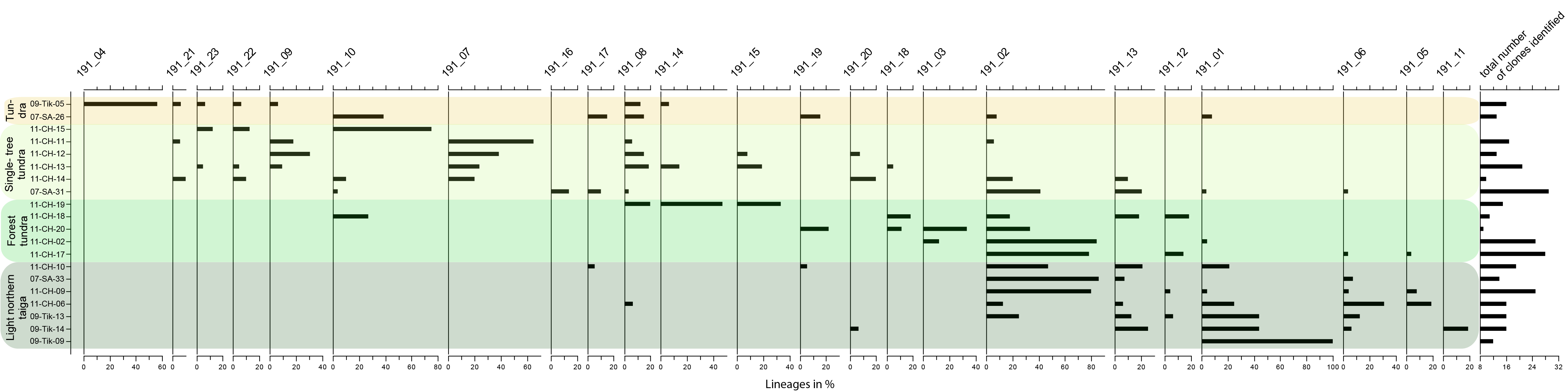
 **Fig. S4.**

B

Frequencies from (A) the *rbcl_a67_01–14* and (B) the *191_01–23* lineages obtained from surface sediments of the investigated lakes. Lakes are arranged according to the vegetation type around the lake.


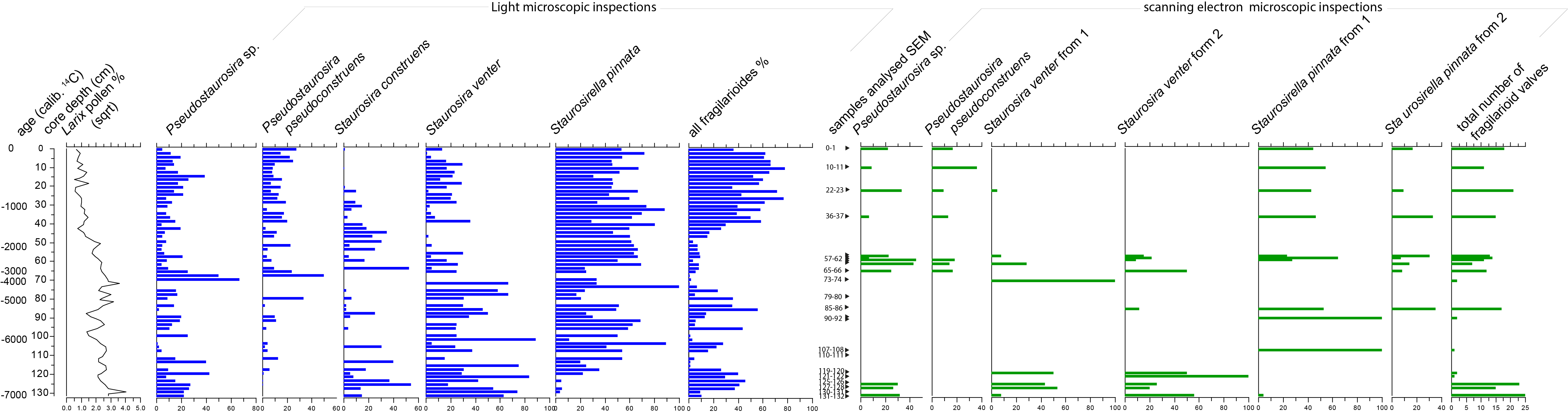


**Fig. S5.**

Down-core (11-CH-12A) variations: Selected diatom counts (species from the Fragilariaceae family) obtained from light microscopic (LM) inspections showing total number of selected counts, percentages of selected fragilarioids, other fragilarioids and other diatoms; and from scanning electron microscopy (SEM) showing total number of valves inspected. Black arrows indicate the samples used for LM and SEM analyses.


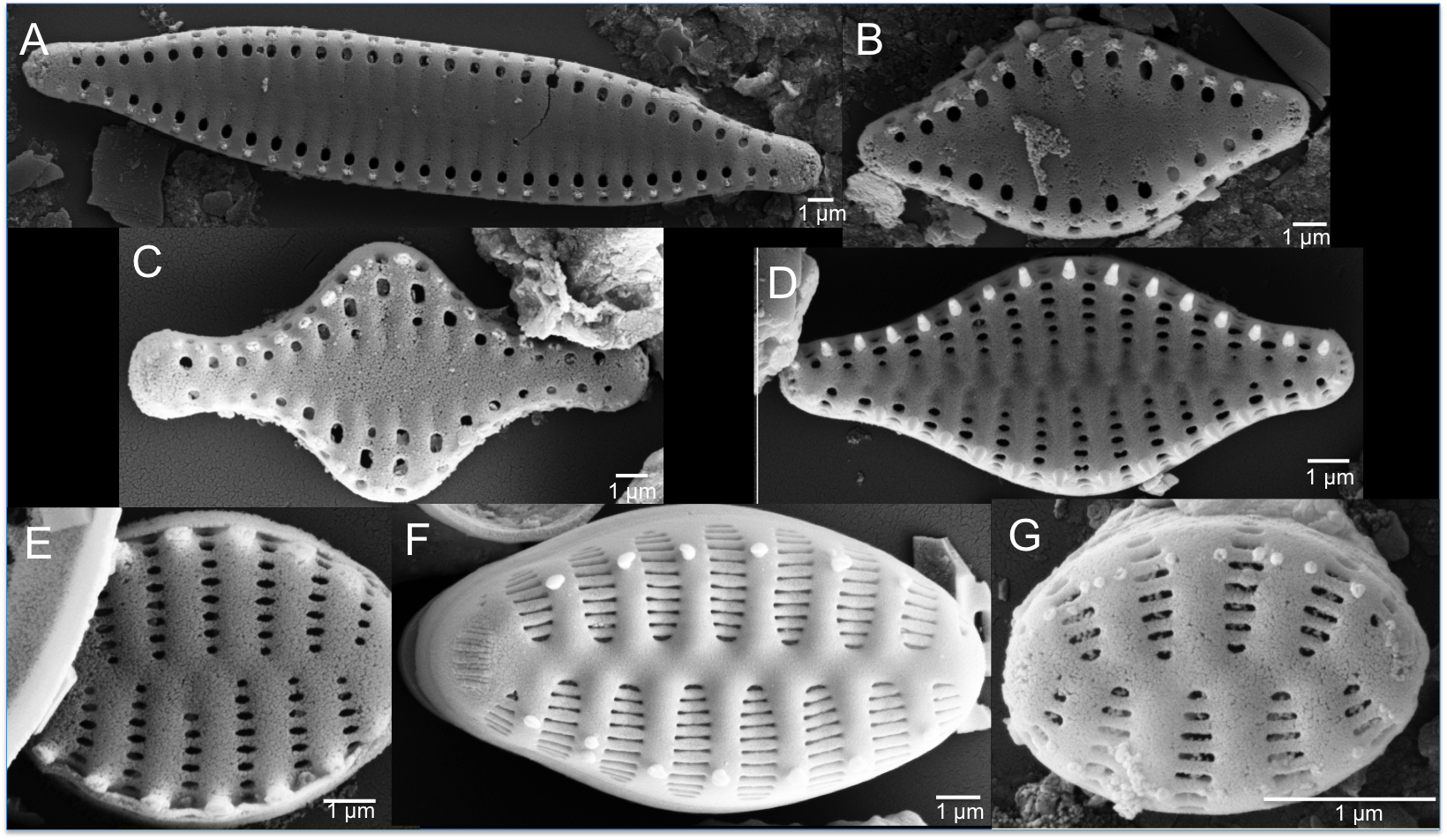


**Fig. S6.**

Scanning electron microscope images A: *Pseudostaurosira* sp. (11-CH-12A-125_126 cm) B: *Pseudostaurosira* sp. (11-CH-12A-125_126 cm), C: *Pseudostaurosira pseudoconstruens* (11-CH-12A-10_11 cm), D: *Staurosira venter* form 1 (11-CH-12A-125_126 cm), E: *Staurosira venter* form 2 (11-CH-12A-65_66 cm), F: *Staurosirella pinnata* form 1 (11-CH-12A-0_1 cm), G: *Staurosirella pinnata* form 2 (11-CH-12A-57_58 cm).

**References**

1. Pestryakova LA, Herzschuh U, Wetterich S, Ulrich M. Present-day variability and Holocene dynamics of permafrost-affected lakes in central Yakutia (Eastern Siberia) inferred from diatom records. *Quatern. Sci. Rev.* **51**, 56-70 (2012).

2. Ficetola GF*, et al.* An in silico approach for the evaluation of DNA barcodes. *BMC Genet.* **11**, 434 (2010).

3. Stoof-Leichsenring K*, et al.* A combined paleolimnological/genetic analysis of diatoms reveals divergent evolutionary lineages of Staurosira and Staurosirella (Bacillariophyta) in Siberian lake sediments along a latitudinal transect. *J. Paleolimnol.*, 1-17 (2014).

4. Stoof-Leichsenring KR, Epp LS, Trauth MH, Tiedemann R. Hidden diversity in diatoms of Kenyan Lake Naivasha: a genetic approach detects temporal variation. *Mol. Ecol.* **21**, 1918-1930 (2012).

5. Hall TA. BioEdit: a user friendly biological sequence alignment editor and analysis program for windows 95 ⁄ 98 ⁄ NT. *Nucleic Acids. Symp. Ser.* **41**, 95-98 (1999).

6. Darriba D, Taboada GL, Doallo R, Posada D. jModelTest 2: more models, new heuristics and parallel computing. *Nat. Methods* **9**, 772 (2012).

7. Clement M, Posada D, Crandall KA. TCS: a computer program to estimate gene genealogies. *Mol. Ecol.* **9**, 1657-1659 (2000).

8. Tamura K*, et al.* MEGA5: Molecular Evolutionary Genetics Analysis Using Maximum Likelihood, Evolutionary Distance, and Maximum Parsimony Methods. *Mol. Biol. Evol.* **28**, 2731-2739 (2011).
